# Metformin protects podocytes by inhibiting the MAPK14–SLC7A11–GPX4 axis and dysregulation of fatty acid metabolism

**DOI:** 10.1007/s00125-026-06821-3

**Published:** 2026-08-03

**Authors:** Shi Qiu, Dandan Xie, Sifan Guo, Zhibo Wang, Ying Cai, Yan Wang, Xiaodan Yu, Yu Guan, Qiqi Zhao, Qiang Yang, Songqi Tang, Wenjie Sun, Yiqiang Xie, Aihua Zhang

**Affiliations:** 1 School of Chinese Medicine, School of Basic Medical Sciences (Key Laboratory of Tropical Translational Medicine of Ministry of Education), International Advanced Functional Omics Platform, Hainan Medical University, Haikou, China; 2 College of Basic Medicine, Graduate School, Heilongjiang University of Chinese Medicine, Harbin, China; 3 Innovative Institute of Chinese Medicine and Pharmacy/Institute of Interdisciplinary Studies, Chengdu University of Traditional Chinese Medicine, Chengdu, China; 4 Department of Nephrology, Traditional Chinese Medicine Integrated Department of Nephrology, The First Affiliated Hospital of Zhengzhou University, Zhengzhou, China; 5 Traditional Chinese Medicine Department, Hainan Health Vocational College, Haikou, China

**Keywords:** Fatty acid metabolism, Ferroptosis, MAPK14/SLC7A11/GPX4, Metformin, Podocyte, Single-cell RNA-seq

## Abstract

**Aims/hypothesis:**

Diabetic nephropathy is a leading cause of end-stage renal disease, with podocyte loss being a critical event in its progression. However, the dominant molecular mechanism driving podocyte loss remains elusive, hindering targeted therapy. We aimed to identify the key pathways of podocyte failure in human diabetic nephropathy and investigate metformin’s therapeutic potential.

**Methods:**

We employed an integrative multi-omics approach, including single-nucleus RNA-seq (snRNA-seq) of 156,043 human kidney nuclei from individuals with diabetic nephropathy and healthy control individuals, *db/db* mouse models of diabetic nephropathy, spatial metabolomics, in vitro podocyte cultures, and targeted urinary metabolomics in individuals with diabetic nephropathy and healthy control individuals.

**Results:**

snRNA-seq identified ferroptosis and fatty acid metabolic dysregulation as the most enriched pathways within podocytes, mechanistically linked to the MAPK14–solute carrier family 7 member 11 (SLC7A11)–glutathione peroxidase 4 (GPX4) axis. In murine diabetic nephropathy models and in vitro, metformin directly inhibited this MAPK14–SLC7A11–GPX4 axis, suppressed podocyte ferroptosis (including restoration of GPX4 and SLC7A11 expression and reduction of p-p38 and lipid peroxidation) and restored compartment-specific renal lipid accumulation as shown by spatial metabolomics. Clinically, we identified and validated a urinary fatty acid signature, with palmitoylcarnitine as a key biomarker (AUC 0.974, calculated from receiver operating characteristic curves), correlating with disease indices including blood glucose, eGFR, serum creatinine and blood urea nitrogen.

**Conclusions/interpretation:**

Our study reveals podocyte ferroptosis to be a central pathogenic event in human diabetic nephropathy, repositioning metformin as a direct ferroptosis inhibitor that preserves podocyte integrity via the MAPK14–SLC7A11–GPX4 axis. Furthermore, we provide a high-performance urinary biomarker, offering a direct translational link toward targeted anti-ferroptosis therapies and non-invasive diagnostics for diabetic nephropathy.

**Graphical Abstract:**

**Figure:**
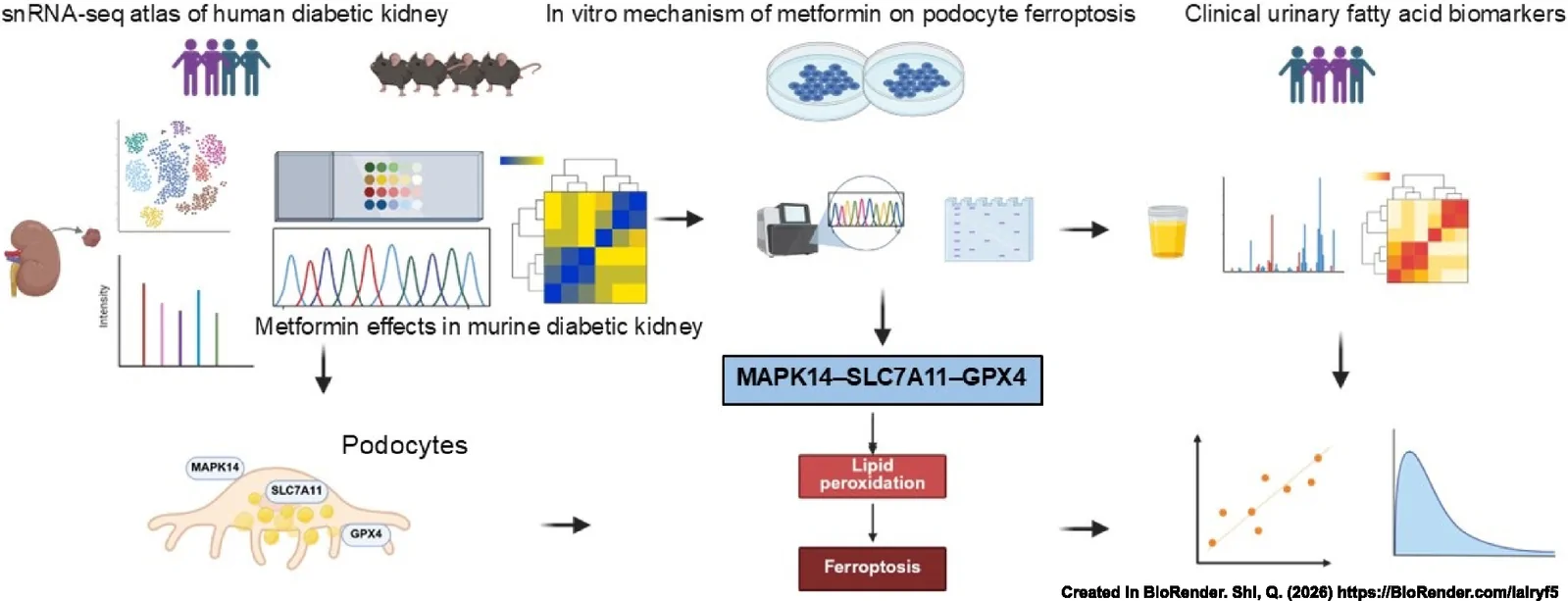

**Supplementary Information:**

The online version contains peer-reviewed but unedited supplementary material available at https://doi.org/10.1007/s00125-026-06821-3.

**Figure:**
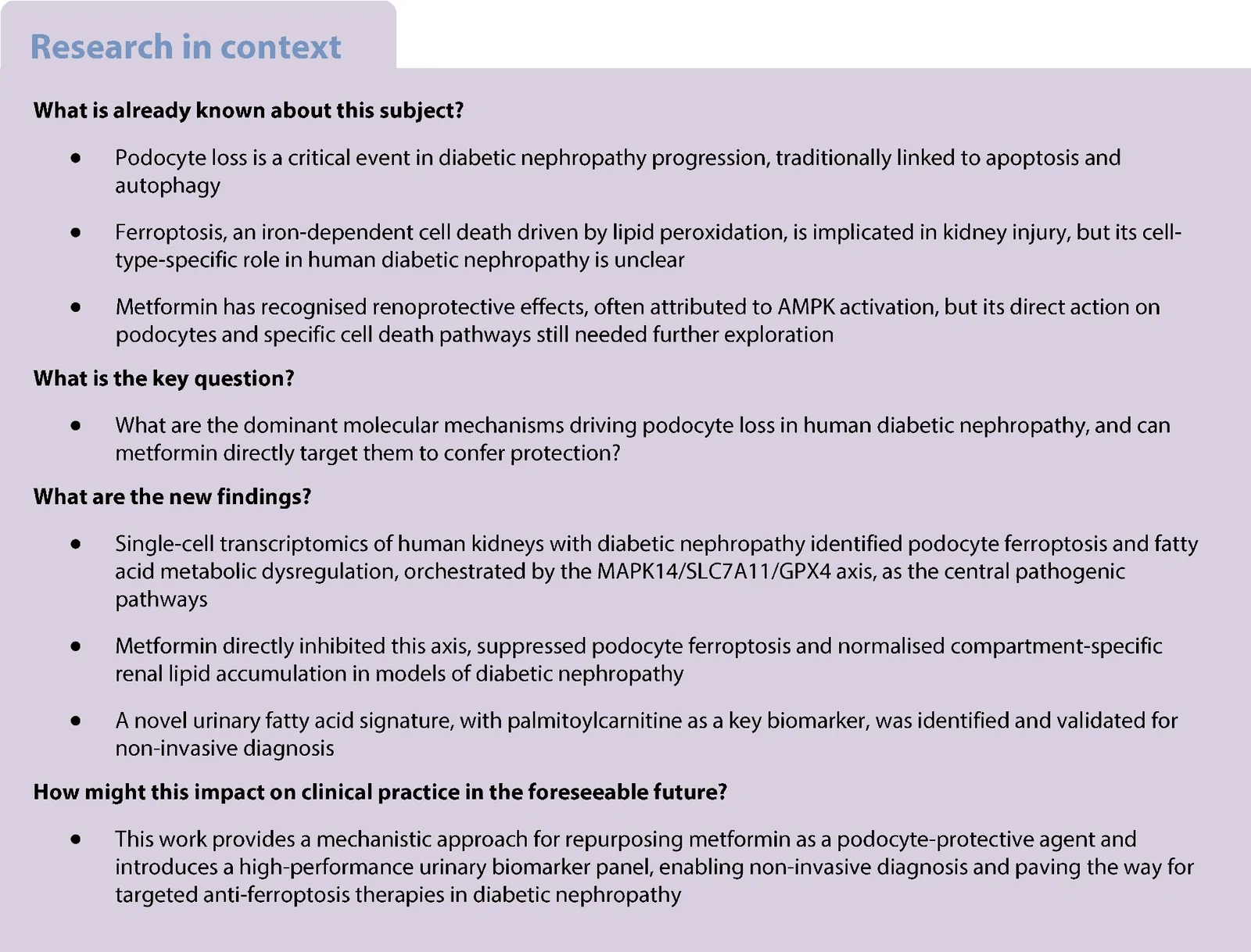

## Introduction

Diabetic nephropathy, the primary cause of end-stage renal disease globally, imposes a severe health burden [1]. Its progression remains incompletely understood and treatment options beyond RAAS blockade are limited, often proving insufficient to halt disease progression [2, 3]. This highlights a critical need to define the cell-type-specific molecular drivers of renal dysfunction. The glomerular podocyte is central to diabetic nephropathy pathogenesis [4]. Podocyte loss correlates with albuminuria and GFR decline [5]. While apoptosis and autophagy have been implicated [6], ferroptosis, an iron-dependent, lipid peroxidation-driven cell death [7], may be a key mechanism. It involves failure of the glutathione-dependent defence system, including solute carrier family 7 member 11 (SLC7A11) and glutathione peroxidase 4 (GPX4) [8]. Concurrent lipid metabolic disturbances can cause lipotoxicity, promoting oxidative stress and dysfunction [9, 10].

Single-cell transcriptomics enables dissection of cellular heterogeneity in complex tissues [11]. Studies have mapped the human kidney landscape in health and disease, identifying injury-susceptible cell subtypes in diabetic nephropathy [12, 13]. Recent spatial multi-omics atlases of the human diabetic kidney have begun to deconstruct this complexity, revealing podocytes as active metabolic-inflammatory hubs orchestrating disease progression [14] and identifying podocyte ferroptosis, driven by dysregulated glycerophospholipid metabolism, as a central pathogenic mechanism in human diabetic nephropathy [15]. These findings not only established a research framework for diabetic nephropathy but also highlighted the therapeutic potential of directly targeting podocyte ferroptosis. Metformin, a first-line glucose-lowering drug, acts partly via AMPK activation [16] but also exhibits AMPK-independent, renoprotective effects [17, 18]. It modulates lipid metabolism and inhibits ferroptosis in various contexts, including via AMPK activation [19–21]. Previous studies have demonstrated that metformin directly protects podocytes by restoring podocalyxin expression [22], reducing oxidative stress-induced podocyte loss [23], and regulating the ZEB2–TG2 axis under hypoxic conditions [24]. However, a direct mechanistic link between metformin, podocyte ferroptosis and lipid dysregulation in diabetic nephropathy remains unestablished.

Here, we aimed to bridge these knowledge gaps using an integrative multi-omics approach. We constructed a single-cell atlas of the human diabetic kidney to determine whether podocyte ferroptosis and lipid dysregulation are dominant pathways. We then investigated whether metformin protects podocytes via the MAPK14–SLC7A11–GPX4 axis, suppresses ferroptosis and normalises renal lipid accumulation in murine models and in vitro.

## Methods

### Human participants and ethical approval

Ethical approval was obtained from the Institutional Review Board of the First Affiliated Hospital of Hainan Medical University (ChiCTR2400087438). The study included 28 individuals with diabetic nephropathy and 25 age- and BMI-matched healthy control individuals (age 58.28±8.95 vs 58.04±9.67 years, *p*=0.925; BMI 25.11±5.13 vs 23.69±2.85 kg/m^2^, *p*=0.210). All comparisons between participants with diabetic nephropathy and healthy control individuals showed statistically significant differences (*p*<0.05) for each clinical variable (electronic supplementary material [ESM] Table 1). Formalin-fixed paraffin-embedded (FFPE) renal tissues were used for snRNA-seq and urine samples were analysed by targeted metabolomics.

### Single-nucleus RNA-seq and transcriptomic profiling

FFPE renal sections (5 µm) from participants with diabetic nephropathy (*n*=3) were placed on Visium HD slides. Spatial barcoding was performed using the Visium HD Gene Expression Kit (10x Genomics). Libraries were sequenced on an Illumina platform, and then processed and analysed as previously described [15].

### Bioinformatic processing and transcriptional analysis

Raw data were aligned to GRCh38 using 10x Genomics Ranger. Expression matrices were analysed in Seurat (R environment). Quality control, normalisation and log-transformation were applied. Highly variable genes were selected for Uniform Manifold Approximation and Projection (UMAP)-based clustering and differential expression analysis (*p*<0.05, |log₂FC|>1). Gene Ontology (GO)/ Kyoto Encyclopedia of Genes and Genomes (KEGG) enrichment was performed via clusterProfiler.

### Animal experiments

Male C57BL/KsJ-*db/m* and *db/db* mice (7 weeks old) were divided into control (*db/m* + saline [154 mmol/l NaCl], *n*=6), diabetic (*db/db* + saline, *n*=6) and treatment (*db/db* + metformin, *n*=6, 65 mg/kg per day) groups. After 4 weeks of oral gavage, kidneys were collected. As detailed in our prior study, *db/db* mice exhibited hyperglycaemia, elevated creatinine/blood urea nitrogen (BUN), and proteinuria [25]. All the procedures were approved by the Institutional Animal Care Committee (no. HYLL-2023-457) of Hainan Medical University.

### Tissue preparation for spatial metabolomics

Mouse kidneys were embedded in 2% CMC, frozen and sectioned at 10 µm using a cryostat. Sections were mounted on ITO slides and stored at –80°C.

### MALDI-MSI acquisition and spatial analysis

Tissue sections were coated with 9-aminoacridine matrix using an HTX TM sprayer. MALDI-MSI was performed on a timsTOF flex in negative ion mode (m/z 150–1200, 30 µm resolution). Data were processed in SCiLS Lab 2024 and annotated via METASPACE (FDR 10%).

### Single-cell RNA-seq analysis of mouse kidney

Mouse kidney single-cell RNA-seq (scRNA-seq) data were processed with Cell Ranger and analysed in Seurat. Low-quality cells and doublets were filtered. Normalisation, clustering and differential expression analysis were performed. GO/KEGG enrichment was assessed via hypergeometric testing.

### Cell culture and treatment

Mouse Podocyte Clone-5 (MPC-5) was purchased from Shanghai Jinyuan Biotechnology Co., (Cat. No. JY013, Shanghai, China). The cell line was authenticated by STR profiling and tested negative for mycoplasma contamination prior to use. MPC-5 mouse podocytes were cultured in DMEM with 10% FBS. At 70–80% confluency, cells were treated for 24 h under the following conditions: normal glucose (NG, control, 5.5 mmol/l); high glucose (HG, 60 mmol/l); HG + metformin (100 µmol/l); or HG + metformin + C16-PAF (5 µmol/l). Cells were lysed with TRIzol and frozen for RNA extraction (*n*=6/group).

### Transcriptome analysis of mouse podocytes

Total RNA was extracted and libraries were prepared using the VAHTS Universal V6 Kit. Sequencing was performed on an Illumina NovaSeq 6000. Reads were aligned to GRCh38 with HISAT2. Differentially expressed genes (DEGs) were identified and functional enrichment (GO/KEGG/Gene set Enrichment Analysis [GSEA]) was analysed [26, 27].

### Western blot analysis of MAPK14/SLC7A11/GPX4

Podocyte lysates were prepared with RIPA buffer (BL504A, biosharp). Proteins (20 µg/lane) were separated by SDS-PAGE, transferred to PVDF membranes and probed with antibodies against p-p38 (14064-1-AP, Proteintech, 1:1000), SLC7A11 (T57046, Abmart, 1:1000), GPX4 (67763-1-Ig, Proteintech, 1:2000), LPO (lipid peroxidase, 10376-1-AP, Proteintech, 1:1000) and loading controls. Bands were visualised and quantified with ImageJ.

### Urinary metabolomic analysis in participants with diabetic nephropathy and in healthy control individuals

Urine samples were extracted with 80% methanol containing an internal standard. Metabolites were separated by UHPLC (HSS T3 column) and analysed by Orbitrap Exploris 120 MS in full scan/dd-MS^2^ mode. Data were processed using XCMS (RSD <30%).

### Development of diagnostic signatures

A predictive model was built using urinary metabolomic data from 25 individuals with diabetic nephropathy and 25 healthy control individuals. Diagnostic performance was evaluated by receiver operating characteristic (ROC) analysis (pROC package) and validated on the CNSknowall platform.

### Statistical analysis

Data are presented as mean ± SD. Group comparisons used two-tailed *t* tests. Multivariate statistical analyses of metabolomics data were performed using MetaboAnalyst (version 6.0). For multiple group comparisons, we performed one-way ANOVA followed by appropriate Tukey’s tests. Analyses were performed in GraphPad Prism v10.

## Results

### Single-cell atlas of human diabetic nephropathy identifies podocyte ferroptosis and fatty acid metabolic dysregulation

**Fig. 1:**
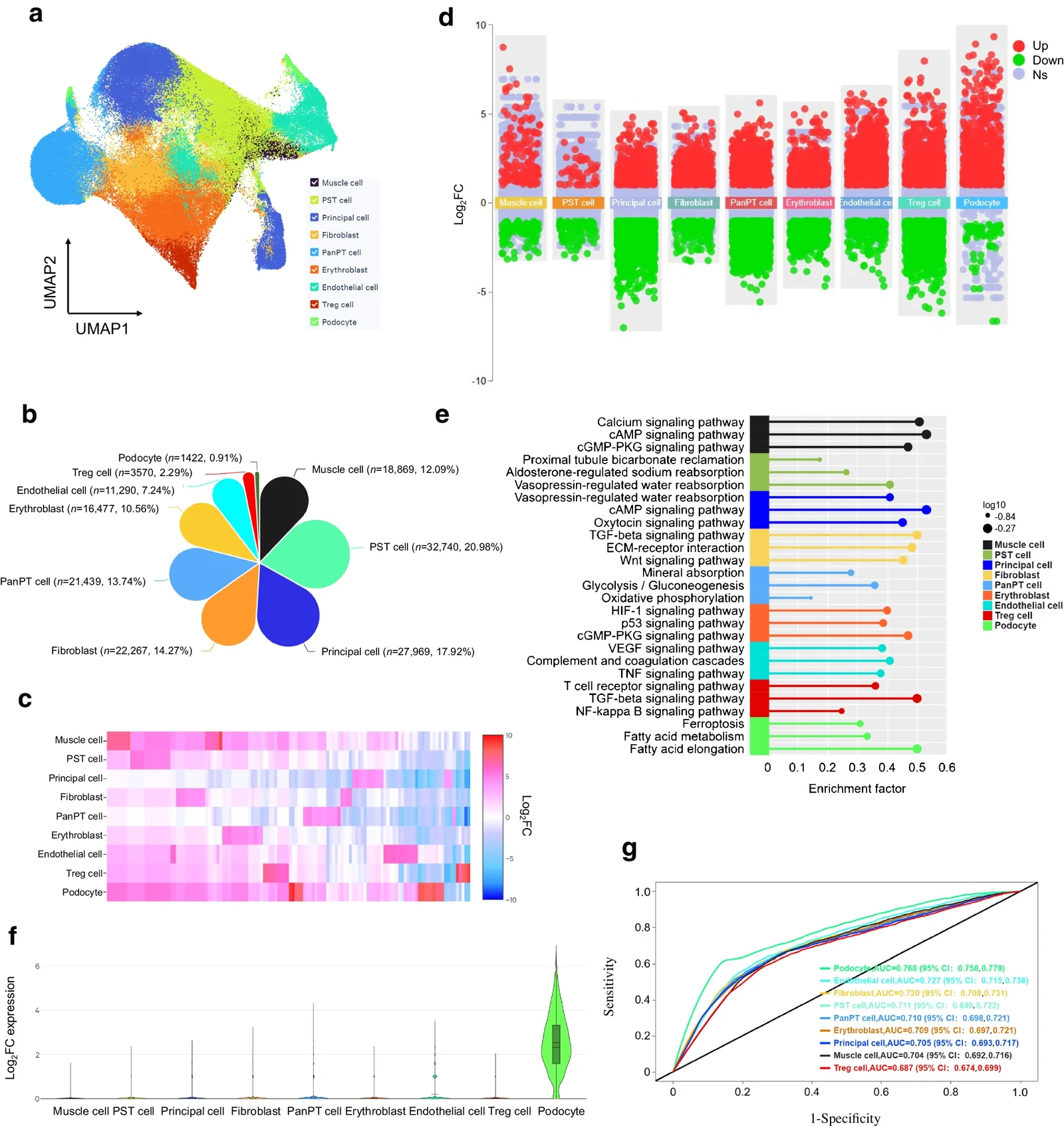
Single-cell transcriptomic profiling of human diabetic kidney identifies podocyte ferroptosis and fatty acid metabolism dysregulation. (**a**) UMAP visualisation of 156,043 high-quality nuclei from renal biopsies of individuals with diabetic nephropathy (*n*=3), annotated to reveal nine major renal cell types (muscle cell, proximal straight tubular cell, principal cell, fibroblast, pan proximal tubular cell, erythroblast, endothelial cell, regulatory T cell, podocyte). Podocytes form a distinct cluster, exhibiting a unique transcriptional state in diabetic nephropathy. (**b**) Petal plot illustrating the relative abundance of each major renal cell type, highlighting compositional changes in the diabetic nephropathy renal ecosystem. (**c**) Heatmap plots showing cell-type-specific expression patterns of representative DEGs across nine major renal cell types, underscoring podocyte-specific transcriptional dysregulation. (**d**) Volcano plot of DEGs across all renal cell types in diabetic nephropathy, highlighting genome-wide transcriptomic alterations. (**e**) Cell-type-specific KEGG pathway enrichment analysis. Podocytes show significant enrichment in ferroptosis and fatty acid metabolism pathways, revealing key mechanistic underpinnings of diabetic nephropathy. (**f**) Violin plots validating podocyte identity using canonical markers (*NPHS1*, *NPHS2*, *WT1*, *PODXL*) and revealing considerable transcriptional heterogeneity within the podocyte population in diabetic nephropathy. (**g**) ROC curve analysis demonstrating the superior diagnostic power of the podocyte-specific transcriptional signature for distinguishing diabetic nephropathy. PanPT, pan proximal tubular; PST, proximal straight tubular; Treg, regulatory T

To define the renal cellular basis of diabetic nephropathy, we established a comprehensive single-cell atlas by performing single-nucleus RNA-seq (snRNA-seq) on 156,043 nuclei isolated from kidneys of diabetic individuals and control individuals. Initially, the analysis of this extensive dataset, visualised using UMAP, successfully delineated the renal cellular landscape into nine major types, including podocytes, proximal straight tubular cells and fibroblasts, among others (Fig. 1a). Subsequently, a petal plot illustrating the relative abundance of these renal cell types demonstrated pronounced compositional shifts in the diabetic kidney, thereby identifying a distinct cellular imbalance within the renal system in diabetic nephropathy (Fig. 1b). Furthermore, to investigate the transcriptional changes, genome-wide profiling via a heatmap plot confirmed widespread differential gene expression across all major cell types, illustrating the extensive molecular perturbations (Fig. 1c). Moreover, cell-type-specific expression patterns of representative DEGs, visualised using volcano plots, distinctly underscored that the most severe transcriptional dysregulation occurs specifically within the podocyte population (Fig. 1d). Consequently, this altered cellular terrain of diabetic nephropathy highlights the podocyte as a primary site of molecular injury.

We next performed a detailed functional analysis to uncover the pathogenic mechanisms underlying podocyte injury. Specifically, cell-type-specific KEGG pathway enrichment analysis across the nine major renal cell types revealed distinct functional alterations; for instance, fibroblasts and T regulatory cells were enriched in TGF-β signalling, while pan proximal tubular cells showed involvement in glycolysis (Fig. 1e and ESM Table 2). Most critically, however, this analysis pinpointed podocytes as being uniquely and significantly enriched in the ferroptosis and fatty acid metabolism pathways. In parallel, validation of podocyte identity using violin plots of canonical markers robustly confirmed the cell annotations while simultaneously revealing extensive transcriptional heterogeneity across the podocyte population in the diabetic kidney (Fig. 1f). Importantly, to assess the diagnostic relevance of these findings, ROC curve analysis demonstrated that the podocyte-specific transcriptional signature achieved the highest diagnostic power for distinguishing diabetic nephropathy, significantly outperforming signatures from all other renal cell types (Fig. 1g). Therefore, by integrating these analytical approaches, our single-cell atlas definitively establishes podocyte ferroptosis and associated lipid metabolic dysregulation as central pathogenic events in human diabetic nephropathy, thereby providing a mechanistic foundation for diagnostic and therapeutic strategies.

### High-resolution podocyte analysis reveals the molecular landscape of podocyte dysfunction

**Fig. 2:**
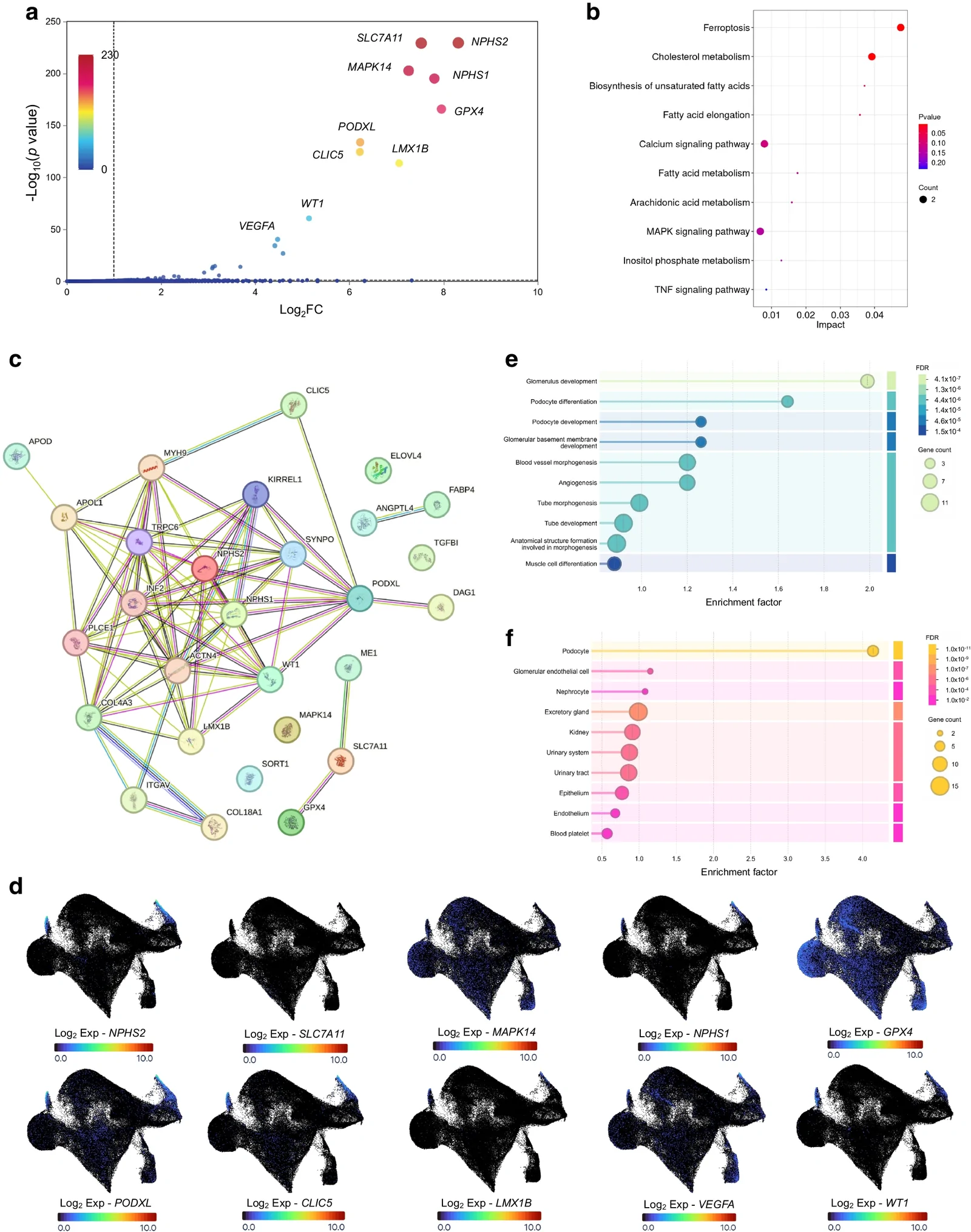
High-resolution dissection of the molecular landscape of podocyte dysfunction in diabetic nephropathy. (**a**) Volcano plot of DEGs specifically within podocytes from individuals with diabetic nephropathy. Key dysregulated genes (*NPHS2*, *SLC7A11*, *MAPK14*, *NPHS1*, *GPX4*, *WT1*, *PODXL*, *VEGFA*, *LMX1B* and *CLIC5*) central to podocyte function are highlighted. (**b**) KEGG pathway enrichment analysis of podocyte DEGs, identifying ferroptosis, fatty acid elongation, biosynthesis of unsaturated fatty acids, and cholesterol metabolism as top altered pathways. (**c**) Protein–protein interaction network of podocyte DEGs, illustrating robust functional connectivity among proteins implicated in cytoskeletal organisation (encoded by *NPHS2*, *NPHS1*, *PODXL* and *CLIC5*), redox balance and ferroptosis regulation (encoded by *SLC7A11* and *GPX4*), and cell survival (encoded by *MAPK14*, *WT1*, *VEGFA* and *LMX1B*). (**d**) UMAP visualisation of the spatial expression patterns of ten key marker genes across podocyte subclusters, revealing state-specific gene expression and functional diversity. (**e**) Functional annotation of podocyte DEGs reveals significant enrichment in biological processes critical for glomerular integrity, including glomerulus development, and podocyte differentiation and development. (**f**) Tissue enrichment analysis confirms that podocyte DEGs are predominantly expressed in glomerular cell types, including podocytes and glomerular endothelial cells. Exp, expression

To define the molecular basis of podocyte failure in diabetic nephropathy, we performed a high-resolution analysis of the podocyte transcriptome. A podocyte-specific volcano plot of DEGs from individuals with diabetic nephropathy revealed profound dysregulation of key genes, including those encoding structural components (e.g. *NPHS1*, encoding nephrin; *NPHS2*, encoding podocin), transcription factors (e.g. *WT1*, encoding Wilms tumour 1) and stress regulators (e.g. *SLC7A11*) (Fig. 2a and ESM Table 3). KEGG enrichment analysis identified ferroptosis and cholesterol metabolism as the most significantly altered pathways, followed by biosynthesis of unsaturated fatty acids and then fatty acid elongation (Fig. 2b and ESM Table 4). Furthermore, a protein–protein interaction network demonstrated functional connectivity among proteins involved in cytoskeletal organisation, redox homeostasis and cell survival, linking dysregulated gene networks to specific pathogenic pathways (Fig. 2c).

We next investigated the cellular heterogeneity and tissue specificity of these perturbations. UMAP visualisation of podocyte marker genes across subclusters revealed considerable transcriptional diversity, with distinct expression patterns of genes such as *NPHS1* and *SLC7A11* defining discrete cellular states (Fig. 2d). GO analysis confirmed significant enrichment in processes critical for maintaining podocyte identity, such as glomerulus development and differentiation (Fig. 2e). Tissue enrichment analysis validated these podocyte-specific DEGs as being predominantly expressed in glomerular cell types, intrinsically linking the molecular signature to glomerular pathology (Fig. 2f). This high-resolution framework connects gene network dysregulation, ferroptosis and cellular heterogeneity to disease progression.

### Metformin preserves podocyte integrity and reverses ferroptosis in a murine model of diabetic nephropathy

**Fig. 3:**
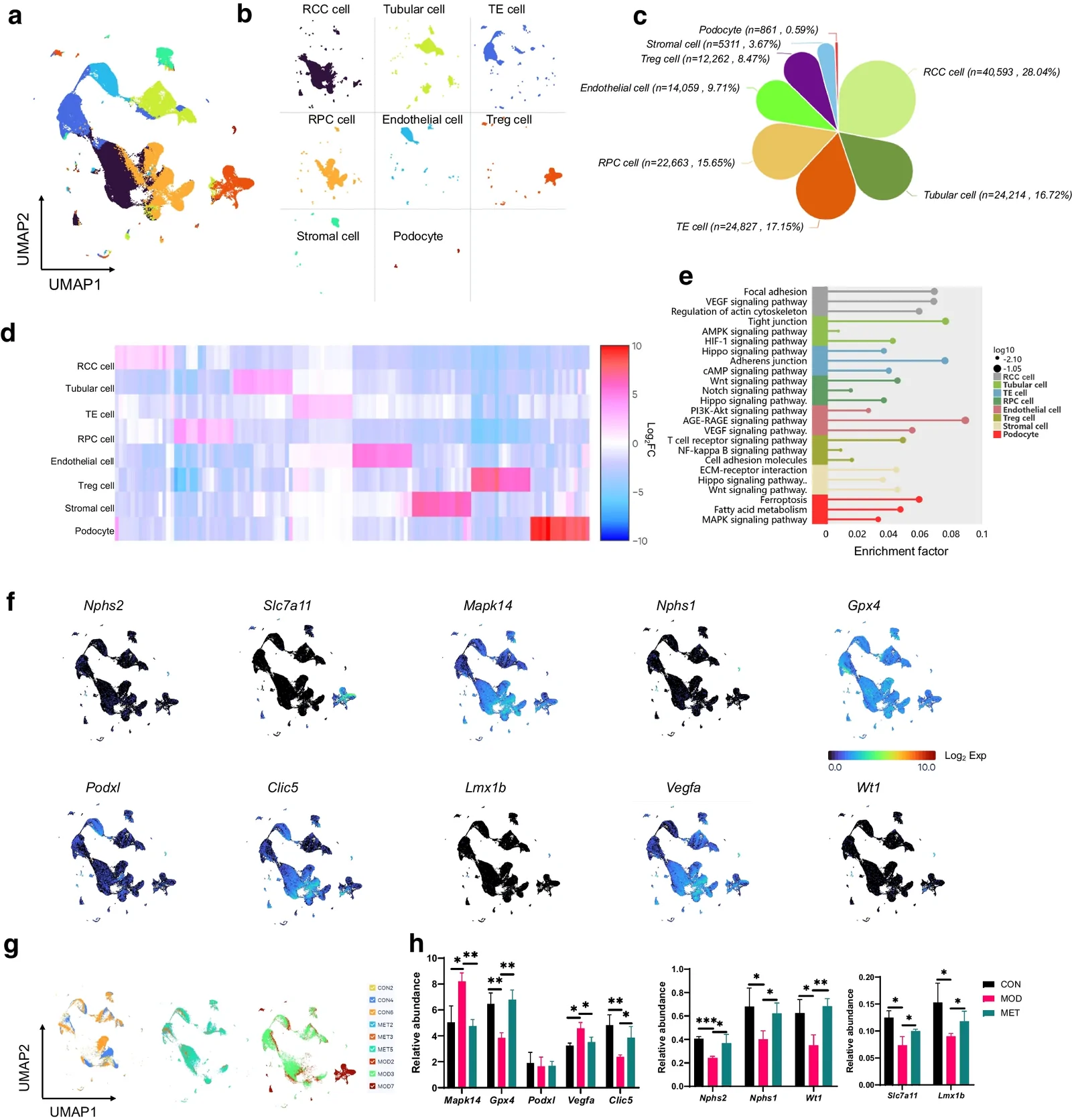
Metformin preserves podocyte integrity and reverses ferroptosis in a murine model of diabetic nephropathy. (**a**) Integrated UMAP visualisation of single-cell transcriptomic data (144,790 nuclei) from control (*db/m*), diabetic (*db/db*) and metformin-treated (*db/db* + MET) mouse kidneys. (**b**) UMAP feature plots showing the distribution of major renal cell types for control, diabetic and metformin-treated groups, including renal corpuscle cells, tubular cells, transitional epithelial cells, renal progenitor cells, endothelial cells, regulatory T cells, stromal cells and podocytes. (**c**) Petal plot displaying the proportional abundance of major cell types for control, diabetic and metformin-treated groups. (**d**) Heatmap plot of DEGs in major cell types from diabetic kidney tissue for control, diabetic metformin-treated mouse kidneys. (**e**) KEGG pathway enrichment analysis shows that metformin specifically reverses diabetes-induced alterations in ferroptosis, fatty acid metabolism and MAPK signalling pathways in podocytes. (**f**) UMAP visualisation of the spatial expression of key marker genes (*Nphs2*, *Slc7a11*, *Mapk14*, *Nphs1*, *Gpx4*, *Wt1*, *Podxl*, *Vegfa*, *Lmx1b* and *Clic5*) from the integrated dataset after metformin treatment. The colour key indicates log_2_-transformed normalised expression levels (log_2_Exp); red, high expression; blue, low expression. (**g**) UMAP plots coloured by MET treatment group, visually emphasising the therapeutic recovery of transcriptional states from control, diabetic and metformin-treated mouse kidneys, respectively. (**h**) Quantitative analysis of key ferroptosis-related genes (*Gpx4*, *Slc7a11* and *Mapk14*) confirming their transcriptional rescue by metformin; calculated as the number of unique molecular identifiers (UMIs) for a given gene divided by the total UMIs per sample. Data are presented as mean ± SD; *p<0.05, **p<0.01, ***p<0.001. CON, control mice; Exp, expression; MET, metformin-treated mice; MOD, diabetic mice; RCC, renal corpuscle; RPC, renal progenitor; TE, transitional epithelial; Treg, regulatory T

To systematically evaluate the therapeutic potential of metformin in diabetic nephropathy, we have established a comprehensive single-cell transcriptomic atlas from murine kidneys. Specifically, we performed an integrated analysis of 144,790 nuclei, including control (*db/m*), diabetic (*db/db*) and metformin-treated (*db/db* + MET) groups, with the UMAP visualisation revealing the global cellular landscape and the impact of intervention (Fig. 3a). Subsequently, UMAP feature plots were used to delineate the distribution of major renal cell types, including podocytes, renal corpuscle cells and tubular cells, thereby providing a detailed reference map for the subsequent analysis (Fig. 3b). Furthermore, a petal plot illustrating the proportional abundance of these major cell types indicated that metformin treatment modulated the renal cellular composition, which was notably altered in the diabetic state (Fig. 3c). To reveal the molecular shifts underlying these changes, a heatmap of DEGs across the major cell types from diabetic kidney tissue highlighted the widespread transcriptomic disruption (Fig. 3d).

Based on this foundational framework, we then focused on elucidating the podocyte-specific mechanisms of metformin’s action. Enrichment analysis identified that metformin partially reversed diabetes-induced alterations in several podocyte pathways, including ferroptosis (Rich Factor=0.0596), fatty acid metabolism (0.0476) and MAPK signalling (0.0334) (Fig. 3e and ESM Table 5). To visually corroborate this rescue, UMAP visualisation of the spatial expression for key marker genes, including structural components (*Nphs2*, *Nphs1*), ferroptosis regulators (*Slc7a11*, *Gpx4*), and signalling molecules (*Mapk14*), displayed a marked restoration of transcriptional patterns towards the healthy state following metformin treatment (Fig. 3f). Moreover, UMAP plots coloured explicitly by treatment group provided the visual evidence illustrating the convergence of transcriptional states in the metformin-treated group towards the control profile (Fig. 3g). Finally, quantitative analysis of key ferroptosis-related genes provided definitive statistical confirmation, showing that metformin significantly rescued the expression of *Gpx4* and *Slc7a11* while suppressing *Mapk14*, thereby transcriptionally reversing the ferroptotic pathway (Fig. 3h). Collectively, our high-resolution data shows that metformin preserves podocyte integrity in a murine diabetic nephropathy model by directly targeting and reversing the core molecular drivers of ferroptosis.

### Spatial metabolomics uncovers fatty acid accumulation in diabetic kidney and its regulation by metformin

**Fig. 4:**
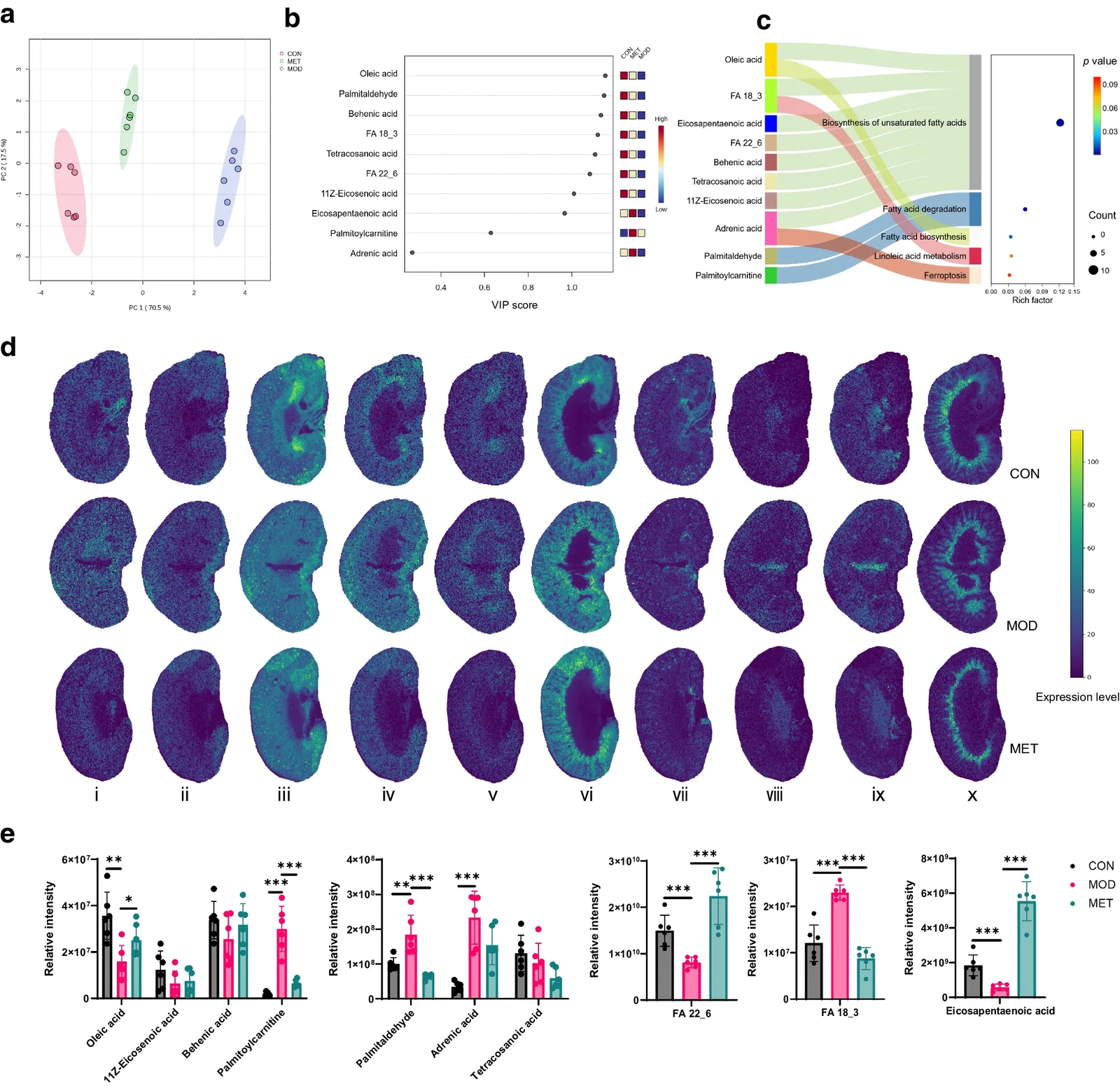
Spatial metabolomics uncovers compartment-specific fatty acid accumulation in diabetic kidney and its regulation by metformin. (**a**) PCA score plot of renal tissue metabolome from control, diabetic and metformin-treated mice, revealing clear metabolic separation between groups. (**b**) VIP plot from the orthogonal partial least squares discriminant analysis model, ranking metabolites by their contribution to group discrimination. Metabolites with VIP > 1.0 are deemed most influential. (**c**) Correlation network diagram illustrating the relationships between dysregulated fatty acid metabolites and their associated metabolic pathways, integrating renal tissue metabolomic and pathway data. (**d**) Spatial distribution maps of key dysregulated fatty acids (ⅰ, palmitaldehyde; ⅱ, FA 18_3; ⅲ, oleic acid; ⅳ, eicosapentaenoic acid; ⅴ, 11Z-eicosenoic acid; ⅵ, FA 22_6; ⅶ, adrenic acid; ⅷ, behenic acid; ⅸ, tetracosanoic acid; ⅹ, palmitoylcarnitine) in kidney sections, obtained by MS imaging, showing distinct cortical and medullary accumulation patterns in diabetes. (**e**) Quantitative analysis of the relative abundance of all significantly altered fatty acids across the three experimental groups, demonstrating that metformin treatment normalises diabetes-induced accumulation. Data are presented as mean ± SD (*n*=6 per group). Abundance values represent total ion current-normalised relative intensities (dimensionless). *p<0.05, **p<0.01, ***p<0.001 (determined by one-way ANOVA followed by Tukey’s post hoc test for multiple comparisons). CON, control mice; FA, fatty acid; MET, metformin-treated mice; MOD, diabetic mice

To characterise the diabetic renal metabolic profile and evaluate the therapeutic impact of metformin, we then performed an integrated metabolomic analysis using multiple analytical platforms. Initially, unsupervised multivariate analysis via principal component analysis (PCA) of mouse renal tissue metabolomes revealed distinct clustering of the control, diabetic and metformin-treated groups, with clear separation along principal components indicating significant global metabolic differences and effectively demonstrating metformin’s partial reversal of the diabetes-induced metabolic profile (Fig. 4a). Subsequently, to identify the key metabolites responsible for this discrimination, the Variable Importance in Projection (VIP) plot from the partial least squares discriminant analysis (PLS-DA) model pinpointed a cohort of metabolites with VIP scores >1.0 (including oleic acid, palmitaldehyde, behenic acid, fatty acid 18_3, tetracosanoic acid, fatty acid 22_6 and 11Z-eicosenoic acid) as the most influential drivers, thereby highlighting the pervasive dysregulation of fatty acid metabolism as a central feature of the diabetic renal metabolome that is modulated by metformin (Fig. 4b and ESM Table 6). Furthermore, a correlation network diagram integrating renal tissue metabolomic and pathway data revealed a coordinated dysregulation of fatty acid metabolism connecting multiple biosynthesis and degradation pathways, while metabolites such as adrenic acid link ferroptosis to lipid peroxidation processes (Fig. 4c).

Spatial distribution maps acquired by MS imaging provided a critical visual dimension, revealing distinct region-specific accumulation patterns of key dysregulated fatty acids (e.g. eicosapentaenoic acid predominantly localised to the renal cortex, while palmitoylcarnitine showed medullary-specific enrichment) (Fig. 4d). Notably, metformin treatment (bottom panels) visibly attenuated these diabetes-induced alterations across both cortical and medullary zones, demonstrating its global moderating effect on renal lipid metabolism (Fig. 4d). To precisely quantify these changes, we conducted a rigorous analysis of significantly altered fatty acids, which revealed profound diabetes-induced dysregulation that was substantially restored by metformin treatment (Fig. 4e). Several key metabolites showed particularly striking changes: palmitoylcarnitine exhibited the most dramatic increase in diabetic kidney, which was nearly completely reversed by metformin; while adrenic acid showed a 6.90-fold elevation that was partially rescued. Similarly, oleic acid decreased to 0.44-fold in diabetes and was restored to 1.58-fold by metformin (Fig. 4e). Consequently, these coordinated normalisations across diverse fatty acid species demonstrate metformin’s efficacy in rectifying the dysfunctional renal lipid metabolism that characterises diabetic nephropathy, providing a profound mechanistic understanding for developing therapeutic strategies.

### Metformin inhibits podocyte ferroptosis via the MAPK14–SLC7A11–GPX4 axis in vitro

**Fig. 5:**
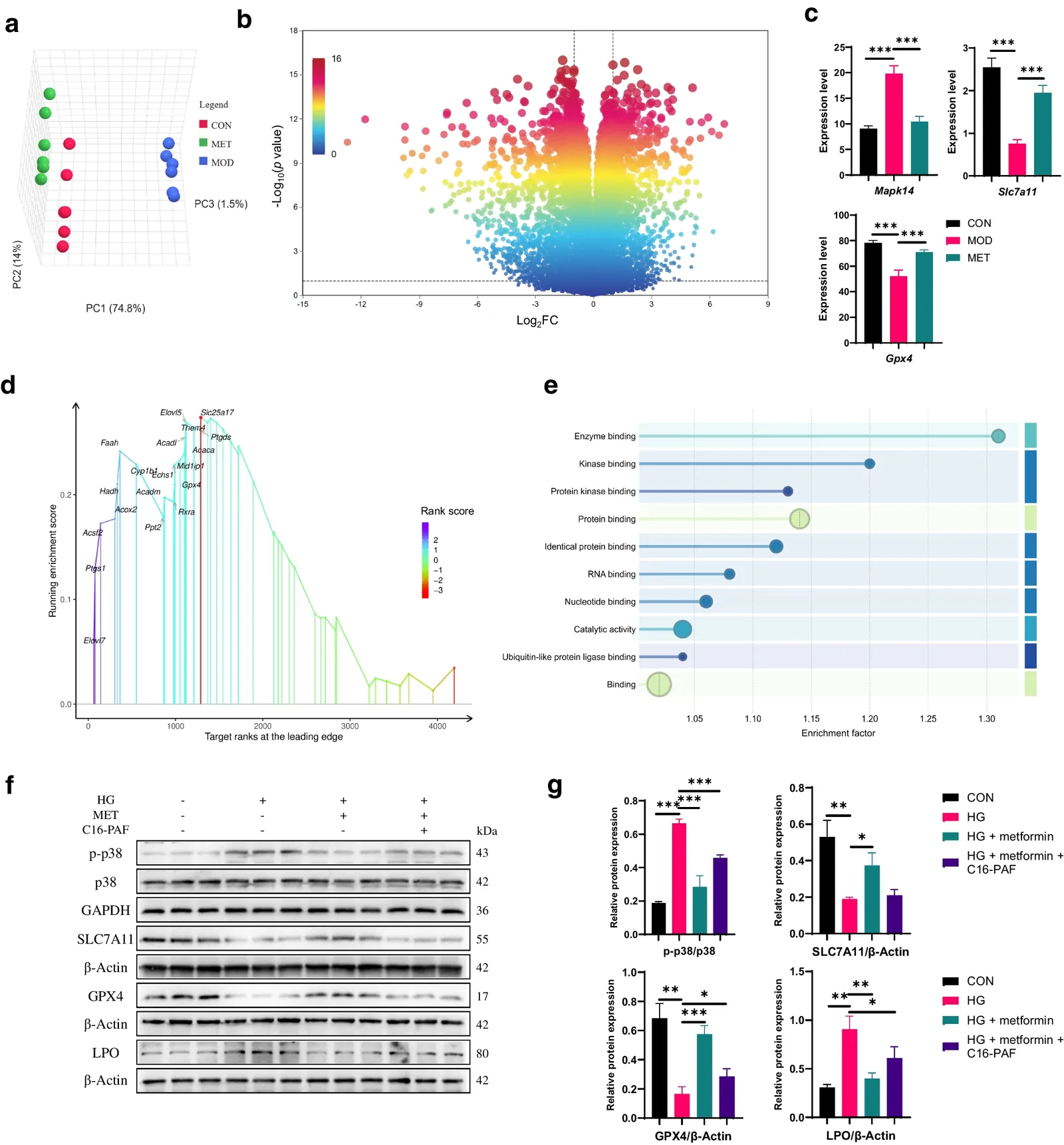
Metformin directly inhibits high glucose-induced podocyte ferroptosis via the MAPK14–SLC7A11–GPX4 axis in vitro. (**a**) PCA of transcriptomic data from MPC-5 podocytes under NG, HG and HG + metformin conditions, showing global transcriptomic rescue by metformin. (**b**) Volcano plot of DEGs in the HG + metformin vs HG comparison, highlighting the reversal of key ferroptosis-related genes. (**c**) Quantitative analysis of expression changes in core ferroptosis regulator genes (*Gpx4*, *Slc7a11*, *Mapk14*), confirming transcriptomic findings. The *y*-axis represents normalised relative expression (dimensionless). (**d**) GSEA plots demonstrating significant enrichment of the ferroptosis pathway signature in metformin-treated podocytes compared with the HG group. (**e**) GO molecular function enrichment analysis of genes rescued by metformin, showing significant enrichment in terms related to protein kinase binding and catalytic activity. (**f**) Representative western blots of key proteins in the ferroptosis pathway (p-p38, SLC7A11, GPX4) and lipid peroxidase (LPO) under different treatments: control, HG, HG + metformin, and HG + metformin + C16-PAF (a p38 MAPK activator). (**g**) Quantification of protein levels from western blots, confirming that metformin’s protection is mediated through inhibition of the p38 MAPK pathway and subsequent restoration of the SLC7A11–GPX4 anti-ferroptosis axis. Relative protein expression was quantified by densitometry and normalised to the loading control (β-actin or GAPDH). Data are presented as mean ± SD (*n*=3). *p<0.05, **p<0.01, ***p<0.001 (determined using one-way ANOVA with Tukey’s post hoc test). CON, NG control condition; MET, metformin-treated podocytes; MOD, HG condition

To delineate the mechanistic basis of metformin’s renoprotective effects, we employed an unsupervised analytical method on MPC-5 podocyte transcriptomic profiles. Initially, PCA of these profiles revealed a clear separation among the NG, HG and HG + metformin groups (Fig. 5a). Importantly, metformin treatment substantially shifted the transcriptional distinct clustering from the HG-distorted state back towards the NG cluster, thereby technically validating its global normalising effect on HG-induced transcriptomic alterations. Subsequently, differential gene expression analysis, visualised by a volcano plot for the HG + metformin vs HG comparison, identified *Mapk14*, *Slc7a11* and *Gpx4* as the significantly regulated genes (Fig. 5b). This finding specifically validates metformin’s transcriptomic rescue effect through this defined pathway in HG-stressed podocytes. Furthermore, quantitative analysis of these key ferroptosis regulators confirmed metformin’s coordinated transcriptomic rescue: *Mapk14* expression was significantly increased under HG conditions (*p*<0.001), which was effectively suppressed by metformin treatment (*p*<0.001); *Slc7a11* showed marked reduction under HG conditions (*p*<0.001) with metformin-mediated recovery (*p*<0.001); and *Gpx4* displayed moderate reduction (*p*<0.001) and partial restoration (*p*<0.001) (Fig. 5c).

Following the identification of this core axis, we next sought to functionally validate the pathway and its regulatory mechanism. GSEA demonstrated significant enrichment of the ferroptosis pathway signature in metformin-treated podocytes, confirming the targeted transcriptional activation of this critical protective program as a key mechanism for countering high glucose-induced injury (Fig. 5d). In parallel, we also identified metformin-rescued genes as being significantly enriched in molecular functions related to protein kinase binding and catalytic activity, highlighting these functional categories as central to its transcriptomic restoration effect (Fig. 5e). To establish causal evidence, the representative western blots of key ferroptosis pathway components demonstrated that metformin upregulates SLC7A11 and GPX4 while suppressing p-p38 and LPO under HG conditions; critically, these effects were reversed by the p38 MAPK activator C16-PAF (Fig. 5f). Ultimately, quantitative analysis consolidated this mechanistic effect, confirming that metformin’s protection is mediated through p38 MAPK pathway inhibition (Fig. 5g). This was evidenced by a 3.55-fold HG-induced increase in p-p38/p38 (*p*<0.01), which metformin reversed to 0.43-fold (*p*<0.01), with subsequent restoration of the SLC7A11–GPX4 anti-ferroptosis axis; SLC7A11 increased from 0.36- to 1.97-fold (*p*=0.011) and GPX4 from 0.24- to 3.49-fold (*p*<0.01), consequently reducing lipid peroxidation (LPO from 2.95- to 0.44-fold, *p*<0.01). All these effects, with the exception of the effect on SLC7A11, were significantly reversed by C16-PAF. Collectively, this study reveals that metformin alleviates diabetic podocyte injury by directly inhibiting the MAPK14–SLC7A11–GPX4 axis, thereby suppressing ferroptosis, a finding that provides a novel mechanistic insight and proposes a promising therapeutic strategy.

### Clinical metabolomic profiling identifies urinary fatty acids as non-invasive biomarkers

**Fig. 6:**
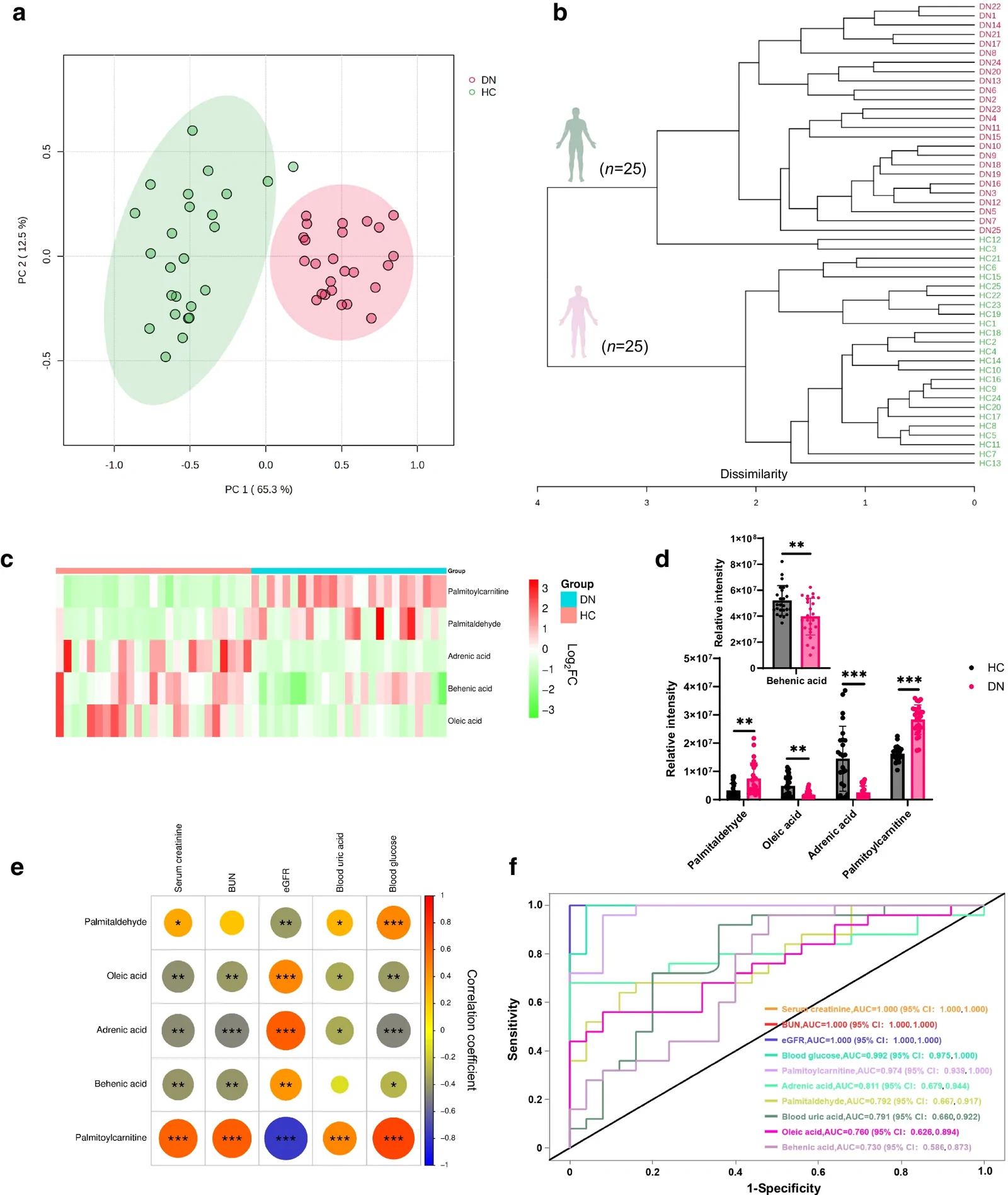
Clinical validation of urinary fatty acid metabolites as non-invasive biomarkers for diabetic nephropathy. (**a**) PCA score plot of urinary metabolomic profiles from individuals with diabetic nephropathy (*n*=25) and healthy control individuals (*n*=25), showing clear separation along the first two principal components, indicative of a distinct urinary metabolic phenotype in diabetic nephropathy. (**b**) Hierarchical clustering analysis of urinary metabolites, including fatty acids and lipid peroxidation products, robustly segregating individuals with diabetic nephropathy from healthy control individuals. (**c**) Heatmap of significantly altered fatty acid metabolites in urine, providing a visual fingerprint of the diabetic nephropathy-specific metabolic disturbance. (**d**) Bar plots displaying the relative abundance changes of key urinary fatty acid metabolites in individuals with diabetic nephropathy compared with healthy control individuals. Abundance values represent total ion current-normalised relative intensities (dimensionless). Abundance values represent total ion current-normalised relative intensities (dimensionless). Data are presented as mean ± SD. (**e**) Pearson correlation analysis between significantly altered urinary metabolites and key clinical parameters of renal function (serum creatinine, BUN, eGFR, blood uric acid, blood glucose), establishing the clinical relevance of the discovered biomarkers. (**f**) ROC curves evaluating the diagnostic performance of individual urinary fatty acid metabolites, highlighting their utility as non-invasive biomarkers for diabetic nephropathy. *p<0.05, **p<0.01, ***p<0.001 (determined using one-way ANOVA with Tukey’s post hoc test). DN, diabetic nephropathy; HC, healthy control

To comprehensively characterise the urinary metabolic phenotype of diabetic nephropathy and validate its clinical utility for non-invasive diagnosis, we performed a detailed clinical metabolomic profiling study. Initially, PCA of urinary metabolomic profiles revealed a clear separation between individuals with diabetic nephropathy and healthy control individuals along the first two principal components, thereby establishing a distinct urinary metabolic phenotype that reflects systemic disruptions and provides a foundation for biomarker discovery (Fig. 6a). Subsequently, unsupervised hierarchical clustering analysis of urinary metabolites, including fatty acids and lipid peroxidation species, generated a clear pattern that effectively segregated the diabetic nephropathy cohort from the control group, thus demonstrating the analytical robustness of these metabolic signatures (Fig. 6b). Furthermore, visualisation of the significantly altered urinary fatty acid metabolites via heatmap analysis created a distinct metabolic fingerprint, technically capturing the specific disturbance pattern in diabetic nephropathy and illustrating the systematic dysregulation of lipid species (Fig. 6c). Ultimately, quantitative analysis of these metabolites revealed profound disturbances in individuals with diabetic nephropathy, with significantly elevated levels of palmitaldehyde and palmitoylcarnitine alongside markedly reduced oleic acid, adrenic acid and behenic acid, thereby establishing a characteristic urinary fatty acid signature that reflects underlying disruptions in mitochondrial β-oxidation and membrane integrity (Fig. 6d).

Beyond identifying these metabolic signatures, we next sought to establish their clinical relevance and diagnostic power. Specifically, Pearson correlation linked urinary fatty acid metabolites to clinical indices of diabetic nephropathy (Fig. 6e and ESM Table 7). Adrenic acid correlated positively with eGFR and negatively with creatinine and BUN; behenic and oleic acids showed significant correlations with renal function parameters; palmitaldehyde correlated positively with blood glucose and negatively with eGFR; palmitoylcarnitine was positively correlated with serum creatinine (*r*=0.6154, *p*<0.001), BUN (*r*=0.6304, *p*<0.001), blood uric acid (*r*=0.4635, *p*<0.001) and blood glucose (*r*=0.7218, *p*<0.001), while showing a strong negative correlation with eGFR (*r*=−0.7722, *p*<0.001). These findings confirm that dysregulated urinary fatty acids reflect both metabolic and functional decline in diabetic nephropathy. Most importantly, ROC curve analysis demonstrated that these urinary fatty acid metabolites exhibit excellent diagnostic performance for diabetic nephropathy. Palmitoylcarnitine achieved exceptional discriminatory power (AUC=0.974), comparable with established clinical markers like blood glucose (0.992) and surpassing blood uric acid (0.791), while adrenic acid (0.811), palmitaldehyde (0.792), oleic acid (0.760) and behenic acid (0.730) all showed significant diagnostic capability (Fig. 6f). Consequently, we identify a definitive urinary fatty acid signature in diabetic nephropathy, which provides profound insights into its metabolic pathology and represents a promising panel of high-performance, non-invasive biomarkers for clinical monitoring, offering complementary information to conventional renal function tests.

## Discussion

This study establishes a mechanistic framework linking podocyte ferroptosis, driven by lipid dysregulation via the MAPK14–SLC7A11–GPX4 axis, to diabetic nephropathy pathogenesis. We reposition metformin as a direct inhibitor of this pathway and derive a non-invasive urinary biomarker signature, translating discovery to clinical application. Our findings significantly extend our previous work, which defined the spatial metabolic-inflammatory landscape of diabetic nephropathy [14], by highlighting the specific cell death program, ferroptosis, as the key executor of podocyte loss.

snRNA-seq of human diabetic nephropathy kidneys pinpointed the podocyte as the epicentre of severe transcriptional dysregulation, uniquely enriched for ferroptosis and fatty acid metabolism pathways [15, 28, 29]. This identifies ferroptosis, characterised by iron-dependent lipid peroxidation [30], as a predominant driver of podocyte loss, with concomitant lipid metabolic dysregulation providing essential substrates for peroxidation [31, 32]. The high diagnostic power of the podocyte signature underscores its central role in determining clinical disease state.

Our molecular dissection revealed interconnected dysfunction. Downregulation of structural genes indicates loss of podocyte identity and integrity [33]. Critically, these defects are functionally linked to core ferroptosis components, creating a vicious cycle in which structural stress impairs redox balance and serves as a critical sensitiser of podocytes to ferroptosis. Observed podocyte heterogeneity likely reflects a spectrum from adaptation to death, explaining variable disease progression [34]. Enrichment in ‘glomerulus development’ processes suggest that diabetic nephropathy corrupts the transcriptional programs maintaining podocyte homeostasis [35].

We delineate a precise axis for metformin’s renoprotection. Beyond AMPK-mediated effects [36], metformin directly reversed the podocyte ferroptosis program by normalising the MAPK14–SLC7A11–GPX4 axis. MAPK14 (p38 MAPK) is a key stress kinase in podocyte injury [37]; metformin suppresses its activation, restoring SLC7A11/GPX4. Abrogation of protection by the p38 activator C16-PAF provides causal evidence. Our prior work showed that ASIV targets upstream glycerophospholipid metabolism [15] and, here, metformin directly inhibits the MAPK14–SLC7A11–GPX4 axis, collectively validating podocyte ferroptosis as a central, targetable diabetic nephropathy pathway. This repositions metformin as a targeted anti-ferroptotic agent for the kidney, a property hinted at in other tissues [38], offering a viable therapeutic strategy alongside direct ferroptosis inhibitors [39]. Notably, our findings are supported by prior work demonstrating metformin’s direct podocyte protection [22–24], and a relevant clinical trial (ClinTrials.gov registration no. NCT06090227) is currently evaluating metformin’s effect on podocyte injury in focal segmental glomerulosclerosis [40].

Spatial metabolomics linked transcriptomic alterations to metabolic topography. Compartment-specific fatty acid accumulation revealed a metabolic alterations map. The dramatic palmitoylcarnitine accumulation suggests impaired mitochondrial β-oxidation, driving lipotoxicity and ferroptosis [31, 41]. Urinary adrenic acid and palmitaldehyde, among other fatty acid metabolites, accumulate due to impaired mitochondrial β-oxidation and lipid peroxidation in diabetic kidneys, reflecting lipid metabolic stress and ferroptosis susceptibility, thereby serving as non-invasive biomarker candidates of podocyte metabolic dysfunction. Metformin’s normalisation of these perturbations connects its known role in enhancing mitochondrial function [42] to suppression of specific cell death in podocytes, explaining how global metabolic disturbances cause precise cellular injury.

The current study has several limitations. First, the human snRNA-seq analysis was performed on a limited number of samples using FFPE tissue, which may affect transcriptome quality. Second, while the *db/db* mouse model reproduces key features of diabetic nephropathy, it may not fully mirror the heterogeneity or chronicity of human disease progression. Third, the urinary biomarker signatures, though promising, requires validation in larger, independent and multi-centre cohorts to confirm its diagnostic and prognostic utility across diverse populations. These limitations highlight areas for future refinement but do not diminish the principal mechanistic insights provided by our integrated approach.

Finally, we translated these insights into the clinic. Urinary fatty acid signature, with palmitoylcarnitine as a potential biomarker, directly reflects renal lipid accumulation. This non-invasive panel correlates with clinical indices and moves beyond albuminuria, a marker of damage [43]. It enables identification of individuals with active podocyte ferroptosis, a process that modulates renal metabolism and may impact responses to ferroptosis [44] or future direct inhibitors. This completes a translational cycle, opening avenues for targeting ferroptosis in diabetic nephropathy and other glomerular diseases, aligning with metabolic reprogramming as a hallmark of kidney disease.

### Conclusion

Our study defines a central pathogenic axis in diabetic nephropathy, linking lipid metabolic dysregulation to podocyte ferroptosis via the MAPK14–SLC7A11–GPX4 pathway. We reposition metformin as a direct inhibitor of this axis, demonstrating its ability to preserve podocyte integrity and normalise renal lipid accumulation. The translation of this mechanism into a sensitive urinary fatty acid biomarker signature completes a translational cycle from discovery to clinical application. This work redefines the molecular basis of podocyte failure and provides a framework for biomarker-driven patient stratification and anti-ferroptosis therapies.

## Supplementary Information

Below is the link to the electronic supplementary material.

ESM Tables (PDF 118 KB)

## Data Availability

The data that support the findings are included within the ESM.

## Abbreviations

BUN: Blood urea nitrogen
DEG: Differentially expressed gene
FFPE: Formalin-fixed paraffin-embedded
GO: Gene Ontology
GPX4: Glutathione peroxidase 4
GSEA: Gene Set Enrichment Analysis
HG: High glucose (60 mmol/l)
KEGG: Kyoto Encyclopedia of Genes and Genomes
LPO: Lipid peroxidase
NG: Normal glucose (5 mmol/l)
PCA: Principal component analysis
ROC: Receiver operating characteristic
scRNA-seq: Single-cell RNA-seq
snRNA-seq: Single-nucleus RNA-seq
SLC7A11: Solute carrier family 7 member 11
UMAP: Uniform Manifold Approximation and Projection
VIP: Variable Importance in Projection
