# Supplementary material for: Metformin protects podocytes by inhibiting the MAPK14–SLC7A11–GPX4 axis and dysregulation of fatty acid metabolism
Source: Diabetologia. 2026 Aug 3;69(11):3312–27. doi: 10.1007/s00125-026-06821-3 (PMC13627274; doi:10.1007/s00125-026-06821-3)
Supplement: Supplementary file 1 [file 125_2026_6821_MOESM1_ESM.pdf]

Electronic supplementary material Table 1. Clinical Characteristics of DN Patients and Healthy Controls

ID	HC	DN	P-value
Number	25	28	-
Female/Male	9/16	8/20	-
Age	58.2800±8.9513	58.0357±9.6704	0.9246
BMI (kg/m2)	25.1132±5.1294	23.6856±2.8537	0.2099
Serum creatinine(μmol/l)	69.1320±13.8220	480.7036±249.3969	0.0000
BUN(mmol/l)	4.6224±1.4146	20.7939±8.5189	0.0000
eGFR(mL/min/1.73m2)	99.6800±12.7106	15.2400±12.7746	0.0000
Blood uric acid(μmol/l)	356.6400±62.0207	457.9600±103.3798	0.0001
Fasting blood glucose(mmol/l)	5.0428±0.5097	8.8607±2.5338	0.0000

Note: BUN: blood urea nitrogen; eGFR: estimated glomerular filtration rate.

DN patients and age-/BMI-matched healthy controls (the Han nationality, China's main nationality,) were recruited from the First Affiliated Hospital of Hainan Medical University.

Electronic supplementary material Table 2. Enriched KEGG pathways across major cell types in DN.

Type	Description	Value
Muscle cell	Calcium signaling pathway	0.51
Muscle cell	cAMP signaling pathway	0.53
Muscle cell	cGMP-PKG signaling pathway	0.47
PST cell	Proximal tubule bicarbonate reclamation	0.17
PST cell	Aldosterone-regulated sodium reabsorption	0.26
PST cell	Vasopressin-regulated water reabsorption	0.41
Principal cell	Vasopressin-regulated water reabsorption	0.41
Principal cell	cAMP signaling pathway	0.53
Principal cell	Oxytocin signaling pathway	0.45
Fibroblast	TGF-beta signaling pathway	0.50
Fibroblast	ECM-receptor interaction	0.48
Fibroblast	Wnt signaling pathway	0.45
PanPT cell	Mineral absorption	0.28
PanPT cell	Glycolysis / Gluconeogenesis	0.36
PanPT cell	Oxidative phosphorylation	0.14
Erythroblast	HIF-1 signaling pathway	0.40
Erythroblast	p53 signaling pathway	0.39
Erythroblast	cGMP-PKG signaling pathway	0.47
Endothelial cell	VEGF signaling pathway	0.38
Endothelial cell	Complement and coagulation cascades	0.41
Endothelial cell	TNF signaling pathway	0.38
Treg cell	T cell receptor signaling pathway	0.36
Treg cell	TGF-beta signaling pathway	0.50
Treg cell	NF-kappa B signaling pathway	0.25
Podocyte	Ferroptosis	0.31
Podocyte	Fatty acid metabolism	0.33
Podocyte	Fatty acid elongation	0.50

Note: The ‘Value’ column represents RichFactor, calculated as the ratio of observed gene count to expected gene count in each pathway.

Electronic supplementary material Table 3. Screening of differentially expressed genes in DN podocytes using volcano plot.

name	log2FC	Pvalue
NPHS2	8.3138	0.0000
SLC7A11	7.5282	0.0000
MAPK14	7.2598	0.0000
NPHS1	7.8055	0.0000
GPX4	7.9572	0.0000
WT1	5.1409	0.0000
PODXL	6.2305	0.0000
VEGFA	4.4748	0.0000
LMX1B	7.0583	0.0000
CLIC5	6.2230	0.0000

Electronic supplementary material Table 4. Enriched pathways in human podocyte DEGs.

Pathway name	P value	Impact
Ferroptosis	0.0038	0.0476
Fatty acid elongation	0.0605	0.0357
Biosynthesis of unsaturated fatty acids	0.0584	0.0370
Fatty acid metabolism	0.1195	0.0175
Cholesterol metabolism	0.0056	0.0392
Arachidonic acid metabolism	0.1312	0.0159
MAPK signaling pathway	0.1423	0.0067
Calcium signaling pathway	0.1083	0.0079
Inositol phosphate metabolism	0.1599	0.0128
TNF signaling pathway	0.2339	0.0084

Electronic supplementary material Table 5. Cell type-specific KEGG pathway enrichment in db/db mouse kidneys.

Type	Description	Value
RCC cell	Focal adhesion	0.0693
RCC cell	VEGF signaling pathway	0.0690
RCC cell	Regulation of actin cytoskeleton	0.0596
Tubular cell	Tight junction	0.0765
Tubular cell	AMPK signaling pathway	0.0079
Tubular cell	HIF-1 signaling pathway	0.0427
TE cell	Hippo signaling pathway	0.0370
TE cell	Adherens junction	0.0761
TE cell	cAMP signaling pathway	0.0402
RPC cell	Wnt signaling pathway	0.0457
RPC cell	Notch signaling pathway	0.0159
RPC cell	Hippo signaling pathway	0.0370
Endothelial cell	PI3K-Akt signaling pathway	0.0272
Endothelial cell	AGE-RAGE signaling pathway	0.0891
Endothelial cell	VEGF signaling pathway	0.0552
Treg cell	T cell receptor signaling pathway	0.0492
Treg cell	NF-kappa B signaling pathway	0.0093
Treg cell	Cell adhesion molecules	0.0166
Stromal cell	ECM-receptor interaction	0.0449
Stromal cell	Hippo signaling pathway	0.0364
Stromal cell	Wnt signaling pathway	0.0457
Podocyte	Ferroptosis	0.0596
Podocyte	Fatty acid metabolism	0.0476
Podocyte	MAPK signaling pathway	0.0334

Note: The ‘Value’ column represents RichFactor.

Electronic supplementary material Table 6. Significantly Dysregulated Fatty Acid Metabolites in db/db Mouse Kidney.

No.	name	exact_mass	formula	KEGG	KEGG_Pathway	CAS
1	Palmitaldehyde	240.2453	C16H32O	C00517	Lipid metabolism	629-80-1
2	FA 18_3	278.2246	C18H30O2	C06426	Lipid metabolism	506-26-3
3	Oleic acid	282.2559	C18H34O2	C00712	Lipid metabolism	68412-07-7
4	Eicosapentaenoic acid	302.2246	C20H30O2	C06428	Lipid metabolism	25377-48-4
5	11Z-Eicosenoic acid	310.2872	C20H38O2	C16526	Lipid metabolism	5561-99-9
6	FA 22_6	328.2402	C22H32O2	C06429	Lipid metabolism	1024594-51-1
7	Adrenic acid	332.2715	C22H36O2	C16527	Lipid metabolism	28874-58-0
8	Behenic acid	340.3341	C22H44O2	C08281	Lipid metabolism	1193721-67-3
9	Tetracosanoic acid	368.3654	C24H48O2	C08320	Lipid metabolism	1219794-61-2
10	Palmitoylcarnitine	399.3348	C23H45NO4	C02990	Lipid metabolism	2364-67-2

Electronic supplementary material Table 7. Pearson Associations between Urinary Metabolites and Clinical Renal Function Indices.

Metabolites	Clinical parameters	Correlation	P value
Palmitaldehyde	Serum creatinine	0.3230	0.0221
Palmitaldehyde	BUN	0.2397	0.0936
Palmitaldehyde	eGFR	-0.3893	0.0052
Palmitaldehyde	Blood uric acid	0.2815	0.0476
Palmitaldehyde	Blood glucose	0.4666	0.0006
Oleic acid	Serum creatinine	-0.4245	0.0021
Oleic acid	BUN	-0.3888	0.0053
Oleic acid	eGFR	0.4640	0.0007
Oleic acid	Blood uric acid	-0.3274	0.0203
Oleic acid	Blood glucose	-0.3858	0.0057
Adrenic acid	Serum creatinine	-0.4482	0.0011
Adrenic acid	BUN	-0.4764	0.0005
Adrenic acid	eGFR	0.6222	0.0000
Adrenic acid	Blood uric acid	-0.3243	0.0216
Adrenic acid	Blood glucose	-0.4769	0.0005
Behenic acid	Serum creatinine	-0.4025	0.0038
Behenic acid	BUN	-0.4071	0.0033
Behenic acid	eGFR	0.4130	0.0029
Behenic acid	Blood uric acid	-0.1224	0.3971
Behenic acid	Blood glucose	-0.2856	0.0444
Palmitoylcarnitine	Serum creatinine	0.6154	0.0000
Palmitoylcarnitine	BUN	0.6304	0.0000
Palmitoylcarnitine	eGFR	-0.7722	0.0000
Palmitoylcarnitine	Blood uric acid	0.4635	0.0007
Palmitoylcarnitine	Blood glucose	0.7218	0.0000
